# Percentage fat fraction in magnetic resonance imaging: upgrading the osteoporosis-detecting parameter

**DOI:** 10.1186/s12880-020-00423-0

**Published:** 2020-03-17

**Authors:** Rong Chang, Xiaowen Ma, Yonghong Jiang, Dageng Huang, Xiujin Chen, Ming Zhang, Dingjun Hao

**Affiliations:** 1 Department of Medical Imaging, Honghui Hospital, Xi’an Jiaotong University College of Medicine, Xi’an, 710054 China; 2 Department of Orthopedics, Honghui Hospital, Xi’an Jiaotong University College of Medicine, Xi’an, 710054 China; 3 Department of Medical Imaging, First Affiliated Hospital, Medical College Xi’an Jiaotong University, Xi’an, 710061 China

**Keywords:** Osteoporosis, Bone mineral density, Magnetic resonance spectroscopy, Fat fraction; MRI m-Dixon-quant

## Abstract

**Background:**

Osteoporosis (OP) is a systemic metabolic bone disorder identified as an essential health issue worldwide. Orthopedic imaging approaches were commonly used with some limitations. Thus, our study aimed to investigate the diagnostic value of magnetic resonance spectroscopy (1-H MRS) and m-Dixon-Quant in OP.

**Methods:**

A total of 76 subjects were enrolled in the study and bone mineral density (BMD) was measured using quantitative computed tomography (QCT). Then, the subjects were divided into three groups according to BMD: normal control group, osteopenia group and OP group. The following parameters were recorded for each patient: gender, age, height, body weight, waist circumference, and hip circumference. Further, the fat fraction percentage (FF%) values were determined by 1-H MRS and m-Dixon-Quant methods.

**Results:**

In both 1-H MRS and magnetic resonance Imaging (MRI) m-Dixon-Quant, the FF% exhibited a negative correlation with BMD (*P* < 0.05). The FF% value of the OP group was significantly higher than that of the control group (*P* < 0.05). In addition, the FF% value in the m-Dixon scans was positively related to age, while BMD showed a negative linear relationship with age (*P* < 0.0001). Further, females had a significantly higher FF% value compared to males (*P* < 0.01), and height was correlated with BMD (*P* < 0.05) but not with FF% (*P* > 0.05).

**Conclusions:**

MRI investigations especially FF% value in the m-Dixon-Quant imaging system is correlated with OP. Its diagnostic value remains to be demonstrated on a large prospective cohort of patients. Besides, parameters such as age, gender, and height are important factors for predicting and diagnosing OP.

## Background

Osteoporosis (OP) is a systemic metabolic bone disorder that affects more than 200 million people especially the elderly worldwide [1]. It is characterized by reduced bone density, leading to an increase in bone fragility and susceptibility to fractures, most commonly vertebral compression fracture (VCF) [2, 3]. Previous study showed that disability rate of OP would be tripled by 2050 with a heavy economic and social burden for the family and society [4]. OP is associated with increased bone marrow adipocytes and impaired bone formation [5]. The formation of bone, hematopoiesis and osteoclast, regulated the signaling pathway in the process of bone marrow adipocyte formation, which is a potential molecular target for the prevention and treatment of OP.

Since the 1990s, the World Health Organization (WHO) has defined OP based on the BMD determined by dual-energy X-ray absorptiometry (DXA), which has been used worldwide [6]. The current detection of BMD is an important basis for the diagnosis of OP; however, it has some limitations since BMD only reflects a part of bone mass, but not the nature of bone marrow, minerals, organic matter and other ingredients [7]. Studies have shown that BMD is correctly diagnosed in only 10 to 53% of older postmenopausal women [8]. BMD evaluation by DXA also has several known limitations since patients were commonly misclassified as having false-negative diagnoses and subsequently received delayed treatments [9]. However, quantitative computed tomography (QCT) and MRS can be used to quantitatively or semi-quantitatively analyze bone micro-structure and intramedullary lipid of OP. And the changes of bone microstructure and bone marrow biomolecular components can be detected before abnormal BMD. Combined application can comprehensively, quantitatively and noninvasively evaluate the bone quality and predict the bone strength, showing a broad application prospect in the OP diagnosis and efficacy monitoring [10]. Nevertheless, QCT has ionizing radiation and it is limited in differentiating healed from non-healed fractures [11].

Therefore, novel tools were urgently needed to diagnose OP, assess fracture risk, and monitor response to therapy. Magnetic resonance imaging (MRI) is a useful non-invasive tool for acquiring in vivo images without X-ray ionizing radiation. It has been revealed that lumbar spine MRI routinely performed for low back pain can be used as an opportunistic screening tool for OP without additional cost, radiation exposure or patients time, but it needs DXA as a reference standard [12]. MR spectroscopy (MRS) belongs to one of the most useful approaches in MRI, which can differentiate healed from non-healed fractures in the human body [13]. Generally, it is expressed by detecting the resonance frequency between the substance and the reference to obtain a relative value of parts per million, which has been gradually applied to the diagnosis of various diseases including tumors [14]. Previous study of the lumbar spine has indicated that bone density has a negative relationship with bone marrow fat content [15]. The 1H MRS method is the only noninvasive method providing information on the biochemical profile of patient tissues in vivo [16]. Further, the m-Dixon-Quant technique is a novel development of MRI [17]. It arbitrarily selects echoes in the signal-acquisition process, thus effectively shortening the TE time. The m-Dixon-Quant technique combines parallel acquisition technologies such as SENSE and dS-SENSE to enhance the imaging speed, and greatly improves the resolution with minimal noise ratio. This technique was found to be effective at calculating lipid content in vivo [18].

One known mechanism of spinal OP is that the adipogenic differentiation of mesenchymal stem cells (MSCs) is upregulated compared to osteogenic differentiation in spinal marrow. The fat fraction (FF) would increase when this balance is broken, which can be assessed using MRS and m-Dixon-Quant. Thus, we hypothesized that the FF percentage (FF%) could be used to diagnose OP. In order to investigate the diagnostic value of 1-H MRS and m-Dixon-Quant in the evaluation of OP, we combined results of the two methods and proposed a novel diagnostic strategy for clinically quantitative analysis of OP.

## Methods

### Patients

This study was approved by the Ethics Committee of Hong Hui Hospital, Xi’an Jiaotong University, and performed between May 2016 and October 2017. A total of 76 subjects with mean age of 59.18 ± 9.22 years were enrolled, 46 males (mean age 59.17 ± 9.06 years) and 30 females (mean age 59.20 ± 9.61 years) were enrolled in the study. All the subjects had no vertebral fractures previously and were divided into three groups according to the results of BMD measurement: normal control group (18 cases), osteopenia group (30 cases) and OP group (28 cases). The inclusion criteria were as follows: (1) clinical data were complete, routine MRI scan and 1H-MRS test were performed; (2) informed consent was signed. The exclusion criteria were as follows: (1) clinical data were incomplete; (2) patients with tumors (3) with other metabolic and endocrine diseases. The following parameters were recorded for each patient: gender, age, height, body weight, waist circumference, and hip circumference. All patients signed informed consent and agreed to participate in the survey.

### BMD measurements

QCT PRO software version 5.1 and Mindways were used for the BMD measurements with synchronous calibration. All the patients underwent QCT scanning of the anterior–posterior lumbar spine (L2–L4) and their BMD values were expressed in mg/cm^3^. PHILIPS 16-slice spiral CT reconstruction was applied to obtain QCT values.

According to the American College of Radiology Guidelines for QCT, all the subjects were divided into three categories based on their BMD results: normal control group (> 120 mg/cm^3^), osteopenia group (80–120 mg/cm^3^) and OP group (< 80 mg/cm^3^) [19].

The BMD values of the L2, L3, and L4 vertebrae were obtained, and the average BMD was calculated using the mean value of L2, L3, and L4. An OP or osteopenia diagnosis was primarily determined in accordance with the average BMD value.

### 1-H MRS examination

Routine MR scans and 1-H MRS scans were performed in all subjects when available. In all cases, 52 cases received the MR scan. The 1.5 T PHILIPS superconducting MR machine (Philips Medical Systems, Best, Netherlands) was used for spectral acquisition. Through conventional MRI plain scanning, as well as sagittal and transverse scanning, the following indexes were set: T2WI/TSE TR 2500, TE 100, two acquisitions, FOV 160*302*57 mm, layer thickness 4 mm, spacing 0.8 mm, matrix 180*237; and T1WI/TSE TR 400, TE 8, FOV 160*299*57 mm, layer thickness 4 mm, spacing 0.8 mm, matrix 160*214. The 1H MRS scan used a single element Point Resolved Spectroscopy (PRESS) sequences for the sagittal and transverse scans. The MRS scan parameters were as follows: TR 2000 ms; TE 42 ms; wave width 1000 ms; excitation frequency 120 times; voxel 15 mm*15 mm*12 mm. The area was reproducible and easy to operate. Thereby, the middle vertebral body was taken as the representative. Based on the previous research [20], we recorded the water peak of the L3 vertebral body displacement around 4.70 ppm and the fat peak between 1.30 and 0.90 ppm.

The FF% values were calculated using the formula: FF% = I_fat_ / (I_water_ + I_fat_) * 100%, where I_water_ or I_fat_ represented the peak of fat or water in the examined substance [21]. This parameter refers to the relative fat signal strength amplitude in relation to the total amplitude (water + fat). After scanning with 1H MRS and m-Dixon-Quant sequences, post-processing software of PHILIPS MR machine automatically calculated I_water_ or I_fat_ values.

### M-Dixon-quant scanning

Meanwhile, the 1.5 T PHILIPS superconducting MR machine (Philips Medical Systems, Best, Netherlands) was used to capture the m-Dixon-Quant images. The conventional settings were identical to the 1H MRS protocols. For the MRI m-Dixon-Quant 3D scan, six phase diagrams were acquired toward three lumbar vertebrae. The medullary cavity was in the central region of the vertebral body, avoiding the cortical bone at the edge of the vertebral body, and a rectangle of 15 mm*15 mm*12 mm was drawn at the center of the L3 vertebral body as the region of interest. Then, we measured on the fat map made by 1 m-Dixon-Quant. The scanning parameters were as follows: TR 7 ms; multi-echo; echo chain 6; NSA 2, flip angle 5°; FOV 300*369*120; voxel 2*2*4; layer thickness 6 mm; spacing 3 mm; matrix 152*184*60; scan mode 3D; and scan time 51 s. The FF% value was directly reported by the PHILIPS superconducting MR machine (with the Philips 3.0tx superconducting MRI scanner software). The target area of the MRI analysis was the same as the area of the BMD measurement point.

### Statistical analysis

All results were presented as mean ± standard deviation (SD). The relationship between pairs of variables was assessed using the linear correlation analysis (Pearson’s R coefficient). Differences between groups were assessed by one-way ANOVA and the difference between males and females was compared using unpaired *t*-tests. *P*-values < 0.05 were considered statistically significant.

## Results

### FF is negatively correlated with bone density

**Fig. 1:**
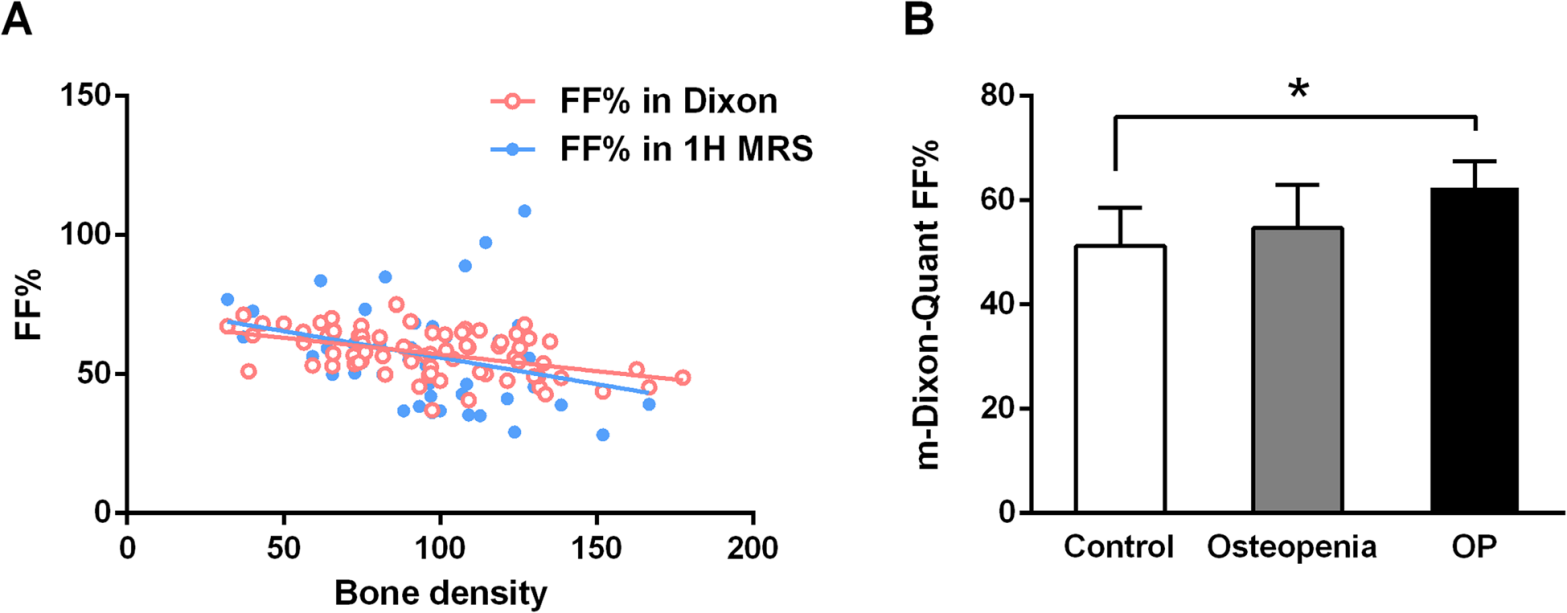
FF is negatively correlated with BMD. **a** The FF% for both 1H MRS and m-Dixon-Quant imaging had a significantly negative correlation with the average bone density value. **b** The patients were divided into three groups: normal control group, osteopenia group, and OP group; the FF% value in the m-Dixon-Quant method was significantly higher in the OP group compared to normal control group. The bar presents the standard deviation; *P < 0.05

**Fig. 2:**
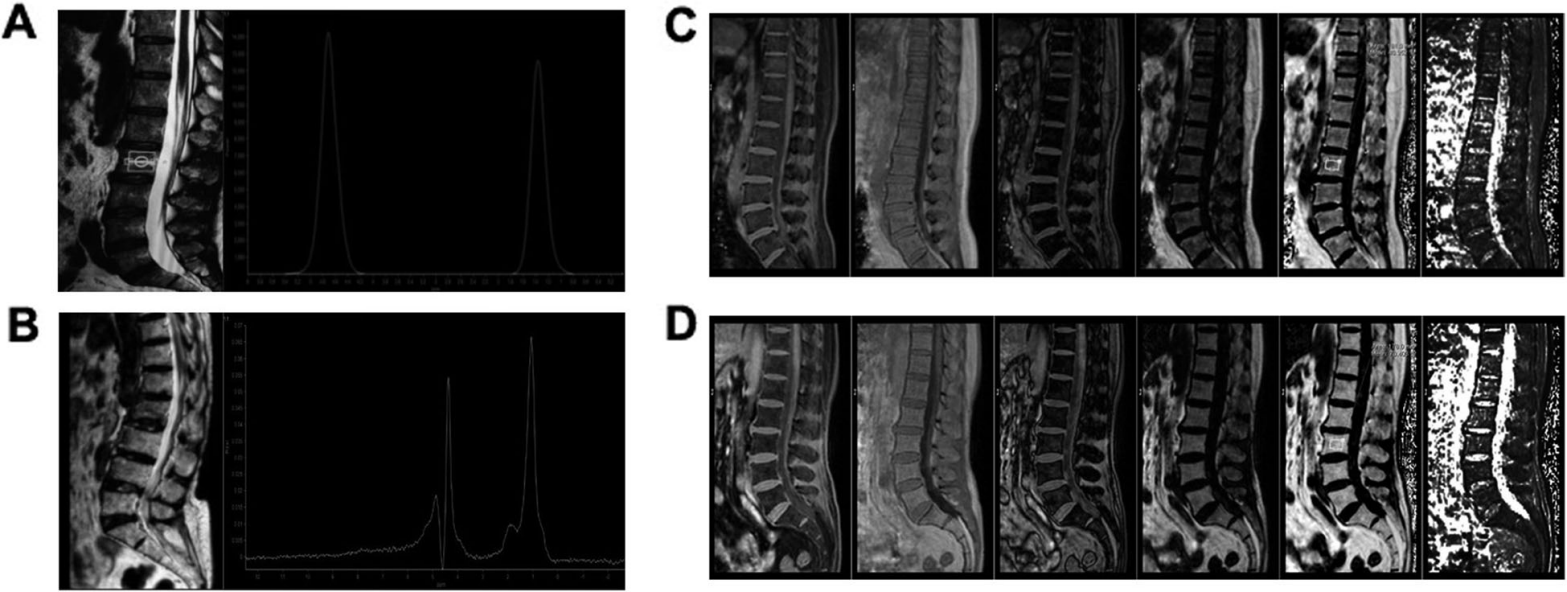
The 1H MRS and m-Dixon-Quant images of two individuals in normal control group and OP group. In the 1H MRS quantitative analysis, the lipid peak of the L2 vertebral body of OP patient (**b**) was significantly increased compared to the normal control (**a**). The same trend was observed for the L3 vertebral body in the m-Dixon-Quant analysis of normal control (**c**) and OP (**d**) cases

First, the parameter FF% was used in both 1H MRS and m-Dixon-Quant methods, and the results showed that the average BMD had a negative relationship with FF% value. As shown in Fig. 1a, the 1H MRS data included 52 cases and a significant non-zero slope was found (Y = –0.1906*X + 75.08; Pearson’s *R*^*2*^ = 0.1046; *P* = 0.0194). Similarly, data in m-Dixon-Quant imaging included 76 cases showed a significantly negative correlation between the average bone density and FF% (Y = –0.1201*X + 69.15; Pearson’s *R*^*2*^ = 0.2200; *P* < 0.001). The patients were divided into three groups, and a consistent conclusion was drawn (Fig. 1b). The FF% value of the OP group was significantly higher than that of the normal control group (m-Dixon-Quant method: FF% = 51.25 ± 7.38 in the control group; 54.70 ± 8.30 in the osteopenia group; and 62.53 ± 5.02 in the OP group; OP versus control, *P* < 0.05). Two individuals in the OP and control groups were presented in Fig. 2. The lipid peaks of the L2 vertebral body in OP patients was significantly increased compared with those in control group (Fig. 2a-b) in the 1H MRS quantitative analysis. The same trend was observed for the L3 vertebral body during the m-Dixon-Quant analysis (Fig. 2c-d).

### Gender and age correlate with BMD and FF% value

**Fig. 3:**
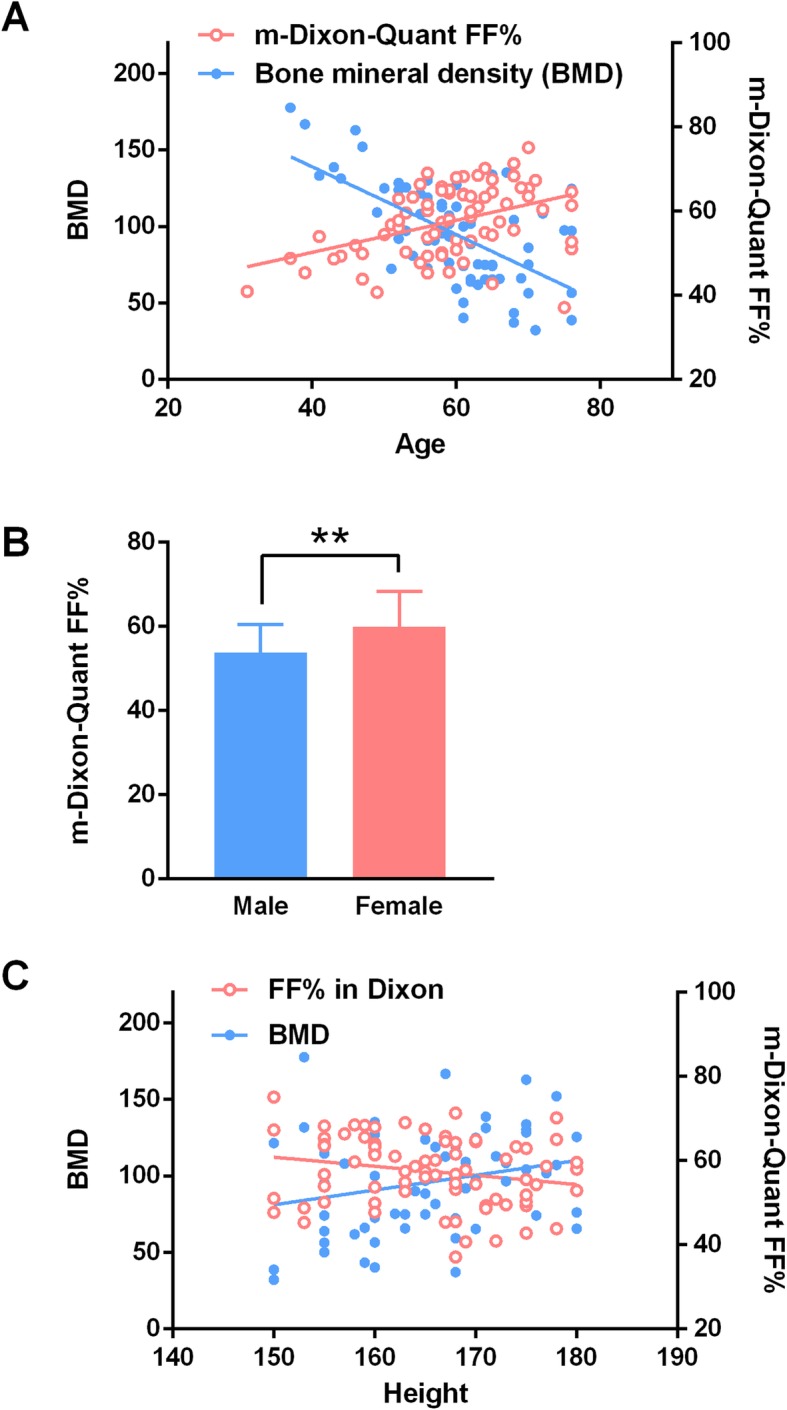
Age, gender, and height correlated with BMD and FF% values. **a** Age largely determined BMD and FF levels **a**. FF% in m-Dixon exhibited a positive correlation with age, while BMD showed a negative linear relationship. **b** Females had a higher FF% level compared to males. The bar presents the standard deviation; **P < 0.01. **c** Height was correlated with BMD, but not FF% (*P* = 0.064)

In addition to FF%, other variables such as the gender, age, height, and weight of each patient were analyzed to probe for more references that may be helpful in the diagnosis. The result showed that BMD and FF% value were largely determined by age (Fig. 3a). The FF% from the m-Dixon scan exhibited a positive correlation with age (F_1,74_ = 16.35, *P* < 0.0001, Y = 0.3807*X + 34.97, Pearson’s *R*^*2*^ = 0.1810), while BMD showed a negative linear relationship (F1, 74 = 42.77, *P* < 0.0001, Y = –2.216*X + 227.6, Pearson’s *R*^*2*^ = 0.3727). This is consistent with the possibility that older adults are at greater risk for developing OP. Further, we found that females had a higher FF% value than males (*t*-test; *P* < 0.01) (Fig. 3b). Interestingly, height was found to be positively related to BMD (F1, 72 = 4.747, *P* < 0.05, Y = 0.9652*X–63.63, Pearson’s *R*^2^ = 0.06185), but not FF% (*P* = 0.064) (Fig. 3c). This finding was reasonable since higher height implied stronger bone density, but the correlation was far below the parameters of gender and age. Further, body weight, waist circumference, and hip circumference were not associated with BMD or FF%. Hence, gender and age correlate with BMD and FF% value, and these parameters may help to diagnose and predict OP when combined with 1H MRS or m-Dixon techniques.

## Discussion

In this study, we applied 1H MRS and m-Dixon-Quant 3D scanning in diagnosing OP and proposed that FF% may reflect the BMD value. We found that both 1H MRS and m-Dixon-Quant imaging could measure bone marrow fat content, which had high clinical value in the diagnosis of OP. Gender, age and height are also associated with OP.

MRI is the only non-invasive method to provide quantitative analysis of tissue metabolism, biochemical environments, and compounds in vivo. Researchers have revealed the relationship between BMD and MRI indexes such as MRI signal intensities and marrow fat in the evaluation of OP [22–24]. However, few studies directly use MRI, especially m-Dixon-Quant 3D scanning, to examine the changes in the L2–4 vertebral body and diagnosis of OP. The m-Dixon-Quant sequence has a short scan time of only 51 s. During the period, the fat score map is intuitively displayed, and the lipid content is quickly quantified without recalculation. However, the m-Dixon-Quant sequence is not configured on all models with the latest development sequence, which is not fully used. We proposed that FF% played a crucial role in MRI-based OP assessments and our results provided a potentially novel strategy for preventing and treating OP that extends beyond BMD observation. Besides, it has been indicated that whether BMD is of predictive value is still controversial. MRI results are correlated with BMD values and provide the early detection of trabecular lesions, fractures, and deformities of the spine [25], while QCT and MRI can image and quantify the three-dimensional structure of trabecular bone [26], hence the combination of these methods may be potential for prevention and treatment strategies. Our results suggest that FF% and BMD permit the structural and metabolic status of vertebrae.

Nevertheless, a team from Italy used MRS and diffusion-weighted MRI to observe heels in normal controls, patients with osteopenia and OP, and found no significant difference in marrow fat content between groups, while the effective internal magnetic field gradient (IMFG), a new MR parameter, held value in OP assessment [27]. This inconsistency with our result may be due to different patient profiles and bone tissues evaluated in both studies.

Bone mass can be estimated by measuring FF using 1-H MRS method. Our study showed that the FF% is positively correlated with age, which is consistent with previous study [28]. The result may due to that the red bone marrow (RBM) changes physiologically to the yellow bone marrow (YBM), resulting in a relative decrease in water content in the red bone marrow and a relative increase in fat content [29]. Our study also found that women are more vulnerable to OP than men, since it has high incidence in postmenopausal women.

We also found that FF had a highly negative correlation with BMD. For patients with OP, RBM gradually transforms into YBM, causing a large number of fat cells to fill in the trabecular bone gap and the loss of dense bone material [30].

In this study, there was a significant difference of FF among the three groups. Through ROC analysis, sensitivity and specificity were high by using FF index to diagnose OP, which was up to 100% in the normal and OP group. FF can be used to diagnose OP since the bone fat content in OP patients is variable, and can reflect the pathological and physiological changes of OP [31].

However, DXA which represents the reference standard was not performed on these patients. Besides, QCT has an increasing fat error with increasing age and the number of cases in this study was small, increasing number of cases were needed to provide further diagnostic information for OP.

## Conclusions

In conclusion, our study indicates that FF% is related to age and BMD. The diagnosis of OP by FF% has high sensitivity and specificity. The application of 1H MRS technology provides a new direction for evaluating and preventing osteoporosis but its diagnostic value remains to be demonstrated on a large prospective cohort of patients.

## Data Availability

All data generated or analyzed during this study are included in this published article [and its supplementary information files].

## Abbreviations

OP: Osteoporosis
VCF: Vertebral compression fracture
